# Our District Examining Boards: How They Do It

**Published:** 1900-07

**Authors:** 


### Correspondence.

### Our District Examining Boards: How They Do It.

HOUSTON, TEXAS, June 19, 1900.

*Editor Texas Medical Journal:*

It is with much pleasure I have just read your excellent report of the proceedings of the State Medical Association. The members of that Association are clearly using every effort to further the interests of legitimate medicine in this State and are abundantly entitled to the support of the balance of the profession and the attentive consideration of the legislators at Austin.

That they do not receive the support that they deserve is witnessed by what occurred in this city on the 28th of April last. An occurrence that puts to blush the "N. Y. Medical College of San Antonio," and the "Independent Medical College of Chicago," because forsooth, it occurred in the ranks and among men generally considered regular. Before the close of Tulane University Medical College, recently, communication was opened between some members of the Medical Examiners Board of the Ninth Judicial District, Texas, and students of that university. My informant states that the correspondence was started by the board. The purport of the interchange of letters was: that all students wishing to practice medicine in Texas could, by being present on a given date at the Hutchins House, in Houston, and the further payment of $15 receive a certificate to *practice medicine and surgery in the State of Texas.* The appointment was kept and on the 28th of April last some fifteen students from all parts of Texas assembled at the place agreed upon. The students were mostly first course men, and yet each and every one received his certificate, seal and all, and duly signed by W. F. Gibson, M. D., President; J. F. Collier, M. D., Secretary.

The certificate of registration obtained from J. B. Williams, District Clerk of Harris county, would indicate that these gentlemen hail from Leggett, Texas.

When our legislative committee from the State Association goes to the legislature this will be excellent material to show the legislators when they say: "We want to do all we can for you doctors." Here are two doctors anyhow who do not need taking care of. With a larger field they would become "Napoleon's of finance."

S. C. RED, M. D.

---

GALVESTON, TEXAS, June 19, 1900.

*To The Texas Medical Journal:*

Please give the enclosed formulas space in your valuable journal. They are the result of years of trial and *have never failed.*

Will follow from time to time with others (the result of twenty-six years of hospital experience).

I.

**For Diphtheritic and Other Ulcerations of the Throat.—Diphtheria—**

℞ Liquor ferri subsulphatis........... ʒi
Glycerinæ, opt..................... ℥i
Spt. Frumenti...................... ℥i
Aquæ puræ, ad......................℥iv

M. S.: Dose, a half teaspoonful every hour or two, according to severity of the case. Can be used advantageously in form of a spray.

II.

**For Epithelial Cancers—**

℞ Zinci chlorid...................gr. 50
Pulv. sanguinariæ..............gr. 50
Amyli..........................gr. 80
Mellis q. s., ut. ft. paste.

S: Apply to cancer and dress surface with carbolized lint when eschar drops off.

III.

**Miasmatic Fever—**

℞ Quiniæ bi-sul...................gr. 18
Phenacetini.....................gr. 6
Ex. opii........................gr. 1

M. bene: Ft. caps. 6.

S: One every two hours.

Will arrest any fever of miasmatic nature.

Fraternally,

C. H. WILKINSON,
Health Physician, Galveston.

